# Enhancing the accuracy of electroencephalogram-based emotion recognition through Long Short-Term Memory recurrent deep neural networks

**DOI:** 10.3389/fnhum.2023.1174104

**Published:** 2023-10-10

**Authors:** Mohammad Reza Yousefi, Amin Dehghani, Hamid Taghaavifar

**Affiliations:** ^1^Department of Electrical Engineering, Najafabad Branch, Islamic Azad University, Najafabad, Iran; ^2^Digital Processing and Machine Vision Research Center, Najafabad Branch, Islamic Azad University, Najafabad, Iran; ^3^Department of Electrical Engineering, K. N. Toosi University of Technology, Tehran, Iran

**Keywords:** emotion recognition, electroencephalography, deep learning, DEAP dataset, Hurst’s view and statistical features, Dual-LSTM

## Abstract

**Introduction:**

Emotions play a critical role in human communication, exerting a significant influence on brain function and behavior. One effective method of observing and analyzing these emotions is through electroencephalography (EEG) signals. Although numerous studies have been dedicated to emotion recognition (ER) using EEG signals, achieving improved accuracy in recognition remains a challenging task. To address this challenge, this paper presents a deep-learning approach for ER using EEG signals.

**Background:**

ER is a dynamic field of research with diverse practical applications in healthcare, human-computer interaction, and affective computing. In ER studies, EEG signals are frequently employed as they offer a non-invasive and cost-effective means of measuring brain activity. Nevertheless, accurately identifying emotions from EEG signals poses a significant challenge due to the intricate and non-linear nature of these signals.

**Methods:**

The present study proposes a novel approach for ER that encompasses multiple stages, including feature extraction, feature selection (FS) employing clustering, and classification using Dual-LSTM. To conduct the experiments, the DEAP dataset was employed, wherein a clustering technique was applied to Hurst’s view and statistical features during the FS phase. Ultimately, Dual-LSTM was employed for accurate ER.

**Results:**

The proposed method achieved a remarkable accuracy of 97.5% in accurately classifying emotions across four categories: arousal, valence, liking/disliking, dominance, and familiarity. This high level of accuracy serves as strong evidence for the effectiveness of the deep-learning approach to emotion recognition (ER) utilizing EEG signals.

**Conclusion:**

The deep-learning approach proposed in this paper has shown promising results in emotion recognition using EEG signals. This method can be useful in various applications, such as developing more effective therapies for individuals with mood disorders or improving human-computer interaction by allowing machines to respond more intelligently to users’ emotional states. However, further research is needed to validate the proposed method on larger datasets and to investigate its applicability to real-world scenarios.

## 1. Introduction

Emotions constitute a valuable facet of an individual’s character and play a pivotal role in the development and acquisition of virtues (Mullins and Sabherwal, 2018). Emotions hold significant significance in human communication and are manifested through a range of expressive patterns known as emotional labeling. Numerous previous studies have focused on emotion classification, often examining commonly studied general emotions such as happiness, sadness, anger, fear, disgust, and surprise. These emotions are typically depicted on a two-dimensional diagram, with stimulation and arousal serving as key dimensions (Al-Nafjan et al., 2017).

Multiple methods have been employed to discern human emotions, including the analysis of speech patterns and tone of voice (Moriyama and Ozawa, 2003; Zeng et al., 2009). However, it is worth noting that this bodily state can be susceptible to manipulation or imitation (Schuller and Schuller, 2021). Facial expressions and their alterations are commonly utilized for emotion recognition; however, these expressions can be intentionally modified by individuals, posing challenges in accurately discerning their genuine emotions (Aryanmehr et al., 2018; Dzedzickis et al., 2020; Harouni et al., 2022). EEG (electroencephalography) is a technique employed to monitor brain activity through the measurement of voltage changes generated by the collective neural activity within the brain (San-Segundo et al., 2019; Dehghani et al., 2020, 2022, 2023; Sadjadi et al., 2021; Mosayebi et al., 2022). EEG serves as a reflection of the brain’s activity and functioning, and it finds diverse applications, including but not limited to emotion recognition (Dehghani et al., 2011a,b, 2013; Ebrahimzadeh and Alavi, 2013; Nikravan et al., 2016; Soroush et al., 2017, 2018a,b, 2019a,b, 2020; Bagherzadeh et al., 2018; Alom et al., 2019; Ebrahimzadeh et al., 2019a,b,c, 2021, 2022, 2023; Bagheri and Power, 2020; Karimi et al., 2022; Rehman et al., 2022; Yousefi et al., 2022, 2023).

Methods rooted in machine learning (ML), pattern recognition, and data mining have been employed to detect and identify emotions through the analysis of EEG signals (Martínez-Tejada et al., 2020). ML, as a highly effective approach, proves invaluable in identifying, diagnosing, and classifying emotions. It facilitates the discovery of patterns that aid in determining distinct types of emotions (Zhang et al., 2020). Nevertheless, while the accuracy of ML tends to improve as the volume of data increases, there exists a threshold beyond which additional data does not yield significant accuracy improvements (Raftarai et al., 2021). To tackle this challenge, deep learning (DL) algorithms have emerged as a solution. DL-based methods demonstrate that as the data becomes larger and more comprehensive, the accuracy achieved increases correspondingly (Alom et al., 2019; Dargan et al., 2020; Karimi et al., 2021).

DL is a ML method (Shrestha and Mahmood, 2019) that encompasses various architectures. One example is Multilayer Perceptron (MLP) networks, which consist of multiple hidden layers, an input layer, an output layer, and hidden units within each layer. Another notable type is Recurrent Neural Networks (RNN), known for their effectiveness in time series prediction, rapid convergence, and adaptability (Rout et al., 2017). In RNN, the output of the hidden layer is fed back, allowing each neuron in the output layer to establish a feedback connection through a buffer layer. This feedback mechanism significantly enhances the RNN’s capacity for learning, recognition, and pattern generation.

In the RNN architecture, each hidden neuron is linked to a single feedback neuron, characterized by a weight of one. Consequently, the feedback layer captures and represents the previous state of the hidden layer. Notably, the number of feedback neurons corresponds to the number of hidden neurons within the network (Jia et al., 2021). In theory while traditional recurrent neural networks (RNNs), have the potential to generate sequences of any complexity given their size, this is often not the case in practice. RNNs struggle with retaining information from past inputs over extended periods, resulting in limited short-term memory. This limitation hampers the network’s ability to effectively model long-term structures and can lead to instability during sequence generation. To address this challenge, Long Short-Term Memory (LSTM) RNNs were introduced. Unlike traditional RNNs, which overwrite their content at each time step, LSTM RNNs incorporate gates that enable them to selectively retain important information. When an LSTM unit identifies a significant feature in the initial steps of the input sequence, it can effectively preserve this information along a longer pathway. This capability empowers LSTMs to handle long-term dependencies and makes them particularly suitable for serving as buffer units within recurrent networks. By leveraging their long-term memory, LSTMs enhance the network’s capacity to improve predictions by considering past context, even if the understanding of recent history may not be perfect (Yin et al., 2021).

LSTM networks represent an advancement over traditional RNNs, specifically designed to address the challenge of retaining past data in memory. By overcoming the problem of diminishing memory capacity in standard recurrent neural networks, LSTM networks offer improved capabilities for tasks such as classification, processing, and forecasting of time series data with uncertain time delays. The training of LSTM networks involves the utilization of back-propagation, a popular technique for model optimization and learning (Yin et al., 2021).

Numerous studies have been conducted to differentiate between emotions and brain signals. Phase Lag Index (PLI) was introduced as a means of identifying multiband static networks (Liu et al., 2019).

In study by Ozel et al. (2019), a novel approach for event-related analysis was introduced, focusing on the time-frequency analysis of multi-channel EEG signals utilizing multivariate transformation. The study incorporated various classifiers, including Support Vector Machine (SVM), K-Nearest Neighbors (KNN), Decision Tree (DT), and Ensemble Classifier. Similarly (Islam and Ahmad, 2019), presented a method that utilized discrete wavelet analysis to classify emotions in EEG signals with a KNN classifier. Ordonez-Bolanos et al. (2019) developed a method for generating a three-component emotion recognition (ER) method, which combines improved full experimental mode analysis, discrete violet transform, and maximum overlap of the discrete violet transform from the brain signal. To determine the class of the feature set, linear discriminant analysis (LDA) and KNN classifications were used in a cascade architecture. Lee et al. (2019) introduced a database and a new method that leverages neural networks to identify emotions. This study described three approaches for identification including support vector machine and neural network-based methods, as well as a belief neural network. Yang et al. (2019) utilized a convolutional neural network with maximum pooling for feature extraction in ER. They employed a neural network with a 50-layer sedimentary architecture, incorporating both maximum pooling and averaging to generate feature vectors. These feature vectors were combined to form the final feature vector and two LSTM models were used for classification. Shukla et al. (2021) employed an optimal feature selection (FS) method to enhance the accuracy of emotion recognition using EEG data. The selected features were then classified using KNN, SVM, and multi-layer perceptron neural network algorithms. Yang et al. (2020) proposed a novel approach for emotion recognition which utilizes the Fourier transform and Mel energy dynamic spectral coefficients. The feature vectors of each signal were then classified using SVM. Wei et al. (2020) developed an emotion recognition method that combines two convolutional neural networks (CNNs) and RNN. This study described the use of CNN for generating high-level features and another CNN for generating features correlated with each other. Then, LSTM RNN was used for further processing and classification of the feature vectors. AlZoubi et al. (2023) classified naturalistic expression of emotions using brain and environmental signals. Khateeb et al. (2021) proposed a novel method for improving the accuracy of ER using EEG signals by combining various feature extraction techniques. This study revealed the characteristics of EEG signals in both time and frequency domains and a multi-class SVM algorithm optimized with a genetic evolutionary algorithm led to improved performance. In the study by Algarni et al. (2022), a range of statistical and Hurst features was employed for classification. To select the most relevant features, the Binary Gray Wolf Optimizer was utilized. ER was performed using a stacked bi-directional LSTM (Bi-LSTM) model. The primary objective of the classification task was to categorize emotions into three main classes: arousal, valence, and liking.

Despite the significant advancements in EEG-based emotion recognition, it is essential to recognize the persistent challenges that continue to shape this field. Existing research in EEG-based emotion recognition has undeniably made valuable contributions, but it is not without its limitations. One notable limitation is the susceptibility of EEG signals to various sources of noise, including environmental interference and artifacts, which can undermine the accuracy of emotion recognition systems. Furthermore, traditional methods of feature extraction from EEG data may struggle to capture the nuanced and complex emotional states that individuals experience. Additionally, the ability to select the most informative features for classification remains a challenging task. Moreover, modeling temporal dependencies and effectively utilizing long-term contextual information present in EEG data is an ongoing challenge. These limitations collectively underscore the need for innovative approaches to address these issues and enhance the accuracy and efficiency of EEG-based emotion recognition systems. However, the existing literature highlights the potential of DL techniques, such as RNN and LSTM, in achieving high accuracy in data classification. However, a critical challenge lies in selecting appropriate features for training these models. This study aims to enhance the accuracy of ER by leveraging DL approaches and brain signals. By employing an effective feature selection technique and combining various classification methods, both high accuracy in ER and reduced computational complexity can be achieved. The primary objective of this paper is to propose an LSTM-based approach for ER using EEG signals.

The proposed method consists of several steps, including preprocessing, feature extraction, dimensionality reduction, and classification. In the preprocessing step, EEG signals undergo quality improvement and noise removal procedures. Subsequently, time/frequency domain features are extracted from the EEG signals to identify the most relevant and informative features for classification. The classification process is performed using the LSTM method.

This study’s key contributions include the extraction of discriminative features from EEG signals, the introduction of an efficient feature selection method, and the improvement of the LSTM architecture for classification purposes. By addressing the challenges of feature selection and leveraging the power of LSTM networks, this approach aims to enhance the accuracy and effectiveness of ER using EEG signals.

## 2. Deep learning

**Figure 1:**
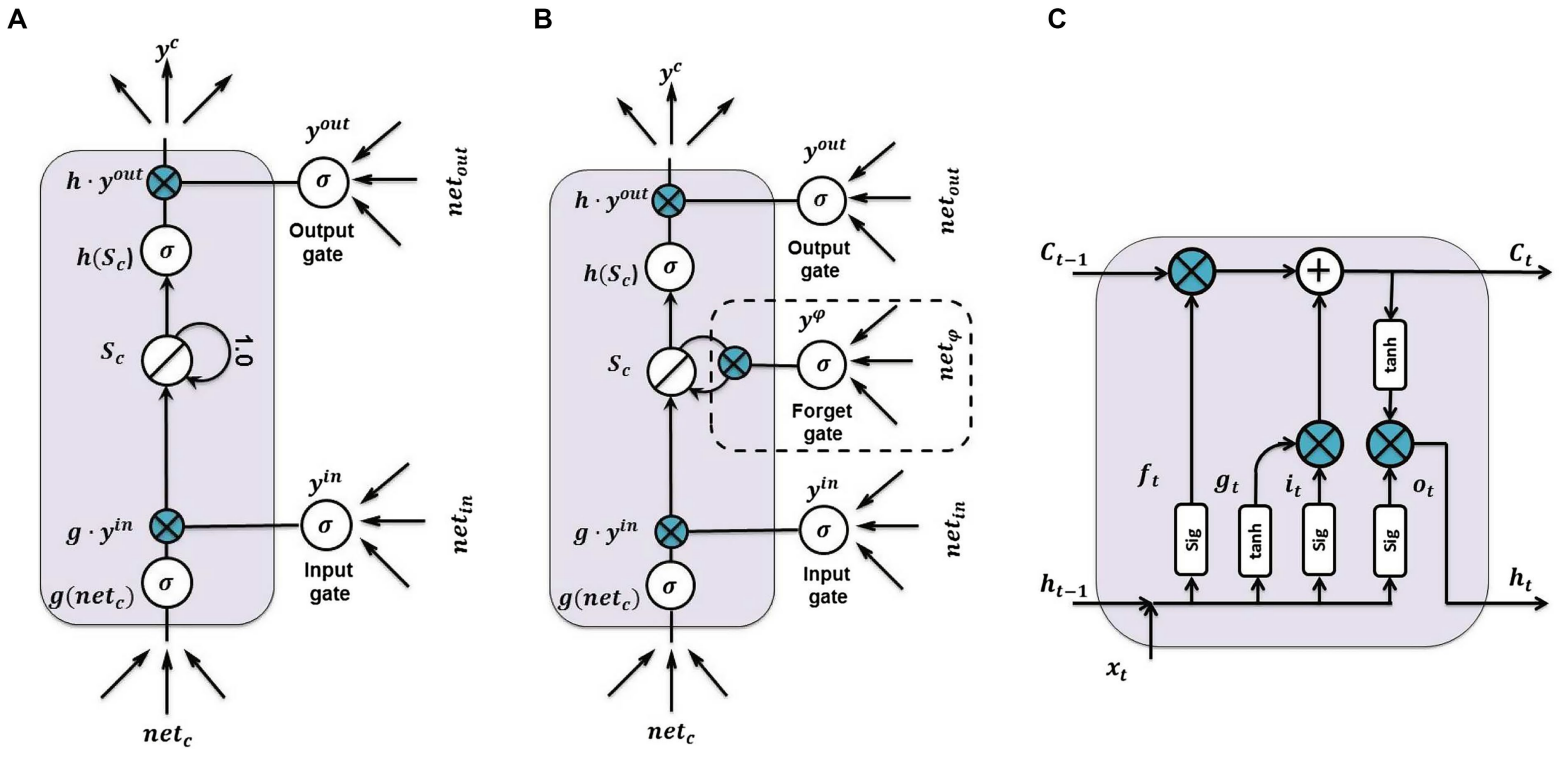
Three LSTM cell configurations: **(A)** Basic LSTM structure with one memory cell and two gates, **(B)** LSTM cell with forgetting gate, and **(C)** updated LSTM model with forgetting gate (Smagulova and James, 2019).

Deep learning utilizes various ML techniques to extract complex patterns and representations from data by employing multiple nonlinear models. The DL workflow consists of two essential stages: training and inference. During the training phase, large quantities of labeled data are examined to reveal their distinctive features. In the inference phase, informed conclusions are drawn, and previously unexplored data is accurately classified or labeled (Leevy et al., 2020). DL, also known as deep structured learning and hierarchical learning, is distinguished by its multi-layered architecture. These layers employ non-linear processing units to efficiently transform and extract significant features from the data (Dargan et al., 2020). In this paper, LSTM has been utilized for ER. Figure 1 illustrates three different configurations of LSTM cells employed in the study (Smagulova and James, 2019). These networks incorporate feedback loops that allow for the retention of information from previous time steps, enabling its persistence within the network. LSTM, a type of recurrent neural network architecture, is specifically designed to improve information storage and retrieval compared to traditional counterparts. Unlike conventional recurrent neural networks that overwrite content at each time step, LSTM networks employ gating mechanisms to selectively retain valuable information. Recurrent neural networks derive their name from the fact that the output of each layer depends on the parameters of preceding layers, granting them the ability to retain and store information from previously observed data. While LSTM is acknowledged as a powerful tool, it still faces challenges such as gradient fading and gradient explosion (Smagulova and James, 2019).

Accurate analysis during the ER process is crucial, particularly in the context of behavioral and mental disorders. However, conducting ER and obtaining precise results can be a challenging task. Previous research papers have highlighted variations in outcomes attributed to various factors, including network experience, environmental conditions, data preprocessing techniques, and classifier selection. As a result, there is an urgent need to develop a more effective methodology to achieve optimal performance. In this regard, this paper presents a novel and efficient approach that leverages the power of DL, specifically utilizing LSTM for ER.

### 2.1. Proposed method

**Figure 2:**
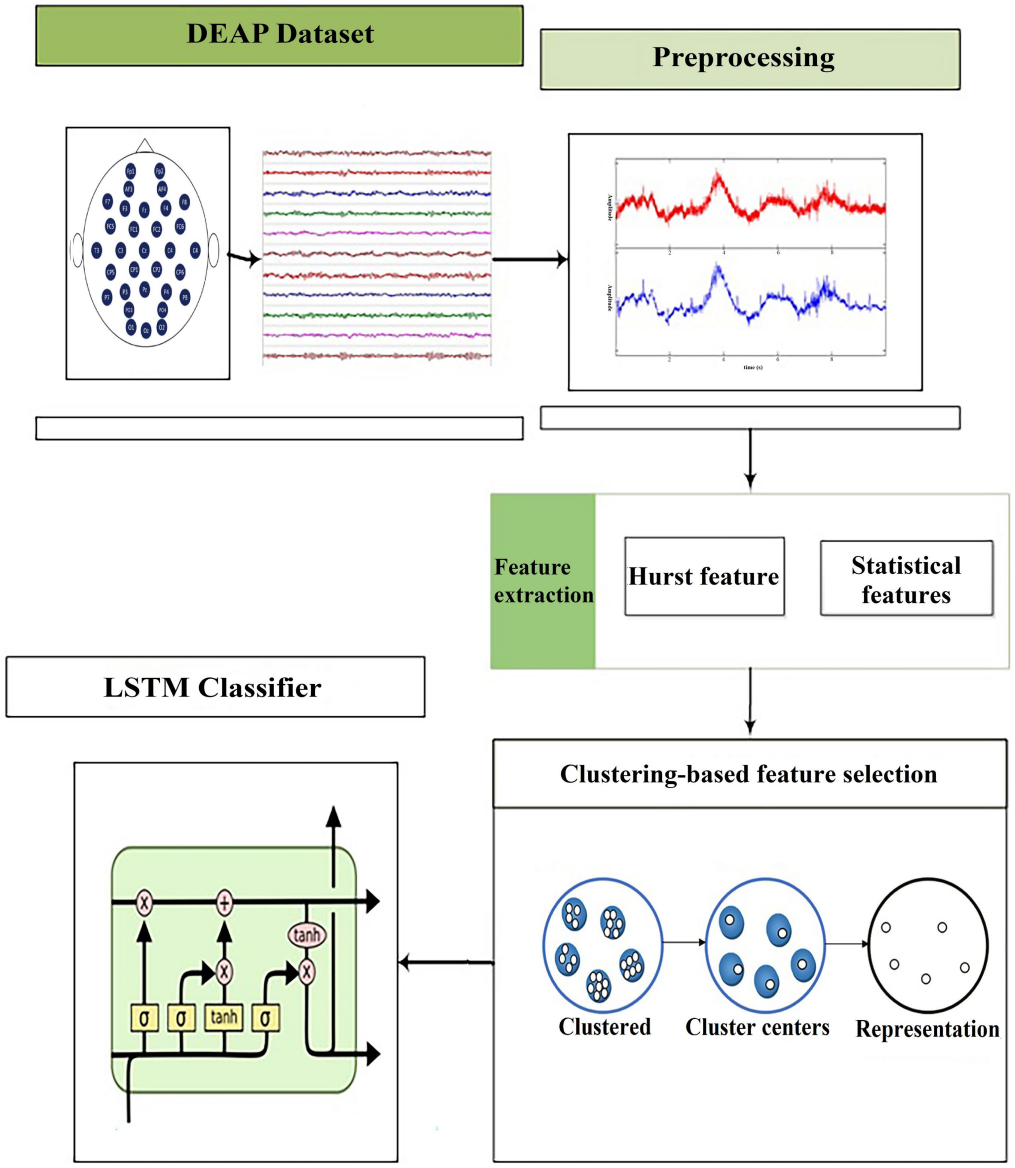
Block diagram of the proposed method (Cho and Hwang, 2020).

This paper focuses on performing ER using EEG signals and employs DL-based classification approach with the LSTM classifier. The LSTM classifier requires key features, such as Hurst features and statistical features, to be extracted from preprocessed input signals. In order to enhance the classifier’s performance, a filter-based FS method will be utilized to choose the most distinctive features for classification. The selected features will then be trained using the proposed LSTM as a DL-based classification approach to recognize different emotions. The proposed method for ER using brain signals is depicted in Figure 2, which illustrates the various stages including preprocessing, feature extraction, dimensionality reduction, and classification. Detailed explanations of these stages will be provided in the following sections.

### 2.2. Preprocessing

**Figure 3:**
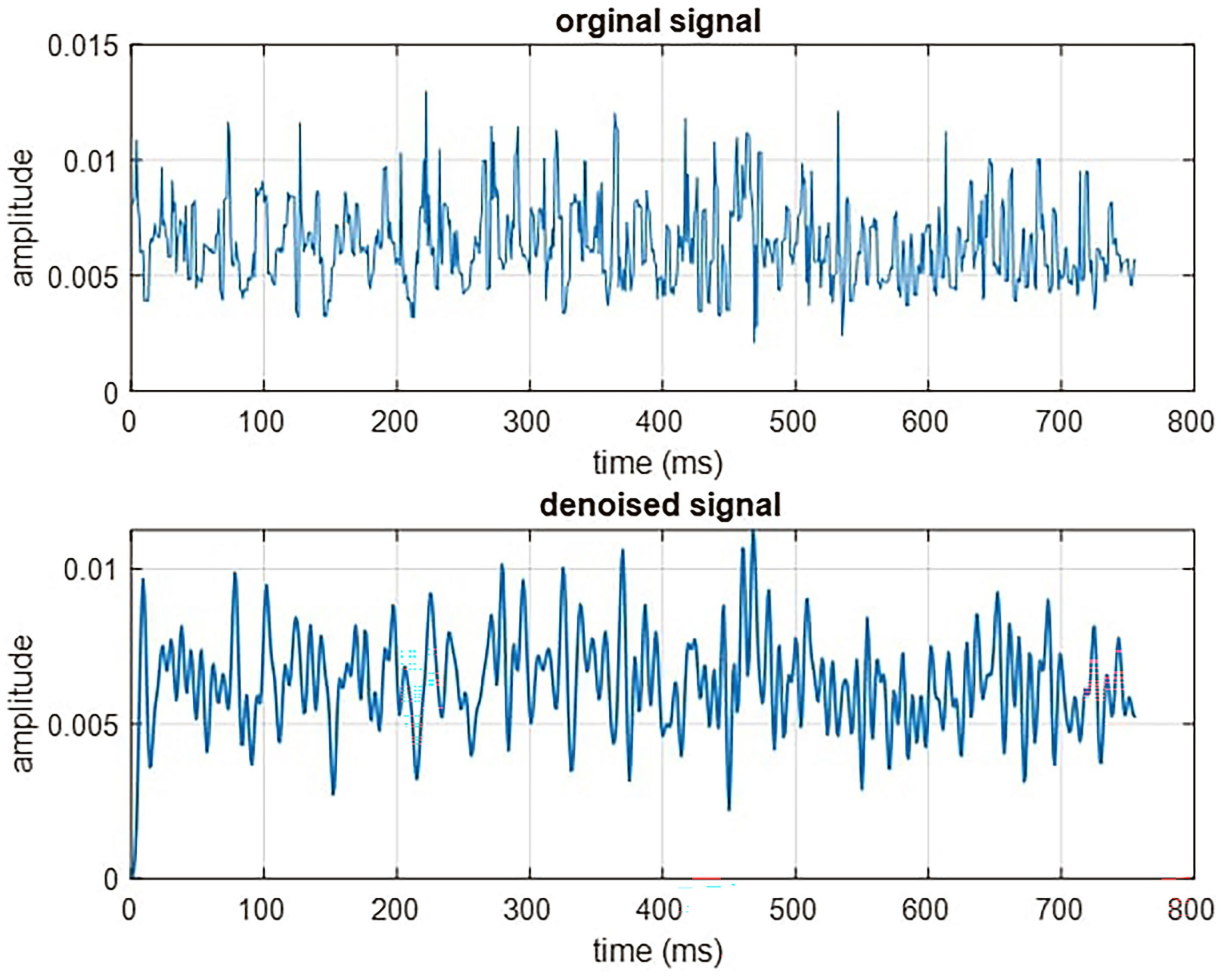
Denoised signal in the proposed method.

Signals can often be accompanied by varying degrees of noise, which may manifest as artifacts and other forms of disturbance in EEG signals. The EEG data from the DEAP dataset underwent preprocessing before, which included artifact removal (specifically EOG), filtering, and down-sampling. Furthermore, a visual inspection was conducted to identify and address any potential artifacts in the data. In addition in this paper, the Empirical Mode Decomposition (EMD) method was employed to eliminate noise from the EEG signal (Huang et al., 1998). The EMD algorithm decomposes the signal x[n] into a set of amplitude-frequency-modulated components, b[n], referred to intrinsic mode functions (IMFs). The EMD technique is an empirical and data-driven technique and in the entire dataset, the number of extremes must be the same as the number of zero crossings or differ by at most one and the mean value of the package defined by the maximum and minimum must be zero. The reconstructed signal x[n] is derived using this approach with m representing the number of samples of a signal. Figure 3 depicts the denoised signal after applying the EMD method.

### 2.3. Feature extraction

Feature extraction plays a crucial role in the effective ER methods based on EEG signals. In this study, feature extraction is done following preprocessing and the generation of noise-free dataset. Feature extraction enables a better understanding of the data and leads to a reduction in computational costs, data storage requirements, and training time. Time and frequency domain features are utilized in this study, and the Hurst view is a notable time domain feature extraction technique employed. In this paper only time Domaine features are extracted.

During the feature extraction process, Hurst’s exponent is employed to measure changes in EEG time series. This feature helps in identifying the presence or absence of long-term trends in sequential one-dimensional signals, such as EEG signal sequences. Equation (1) is used to calculate the Hurst index (Geng et al., 2011; Ebrahimzadeh and Najararaabi, 2016; Algarni et al., 2022).

The Hurst Exponent (HE) quantifies the level of persistence in a time series. It is calculated by taking the cumulative derivative of the mean-centered signal with respect to partial time series of length n, as expressed by the following equation:


(1) Zn=∑j=0nxj−x¯Rn=Znnmax−Zntmin

n this equation, $\bar{x}=\frac{1}{n}\overset{n-1}{\underset{i=0}{\sum }}x\left[i\right]$ is the average of signal with the length of n. HE can be defined as:


(2) $$E\left[\frac{R\left(n\right)}{S\left(n\right)}\right]=\lim_{n\to \infty }Cn^{\mathrm{HE}}$$

In Equation (2), E[.] refers to expectation value and S(n) is the standard deviation of the partial time series.

Due to its non-stationary nature in the time domain, the EEG signal is analyzed using statistical methods to accurately depict its characteristics. A series of statistical features of the EEG signal have been used for ER including skewness (as a degree of asymmetry of a distribution), curve elongation, entropy, mean, variance, signal energy, and Shannon entropy.

### 2.4. Feature selection

This paper introduces the utilization of the FS method for ER, which involves three steps in the repetition and correlation analysis as described below:

1. The feature space is established using the Euclidean distance criterion in this study.
2. The features are classified using clustering methods such as K-means.
3. To obtain the optimal subset of features, an FS method based on the minimum redundancy maximum relevance (mRMR) approach is applied within each cluster. The resulting subset of features is then utilized for classification, with the cluster heads serving as the final features. This approach is thoroughly explained in detail in Ismi et al. (2016).

**Figure 4:**
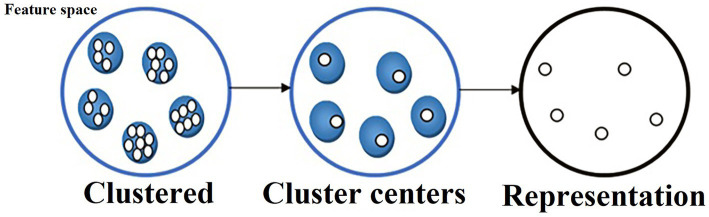
Clustering-based FS method.

The proposed FS method is depicted in Figure 4. Initially, the feature space is clustered, and the resulting cluster centers are regarded as the selected features for the clustered data. The strongly related features are identified as the fundamental features of the Essential attribute, forming the foundation of the conditional features. The optimization process primarily focuses on correlation and repeatability analysis, addressing two key optimization problems. To optimize the selected features, a classic criterion based on repetition or relationship, mRMR and Max (Relevance $\left(\hat{S};C\right)$ ) and Min (Relevance $\left(\hat{S}\right)$ ) will be used.

The mutual information-based mRMR approach only minimizes the mutual information between features. This can improve the classification efficiency of the desired features. With the proposed FS method, the most effective features are selected and then classified using a LSTM model.

### 2.5. Classification with LSTM

**Figure 5:**
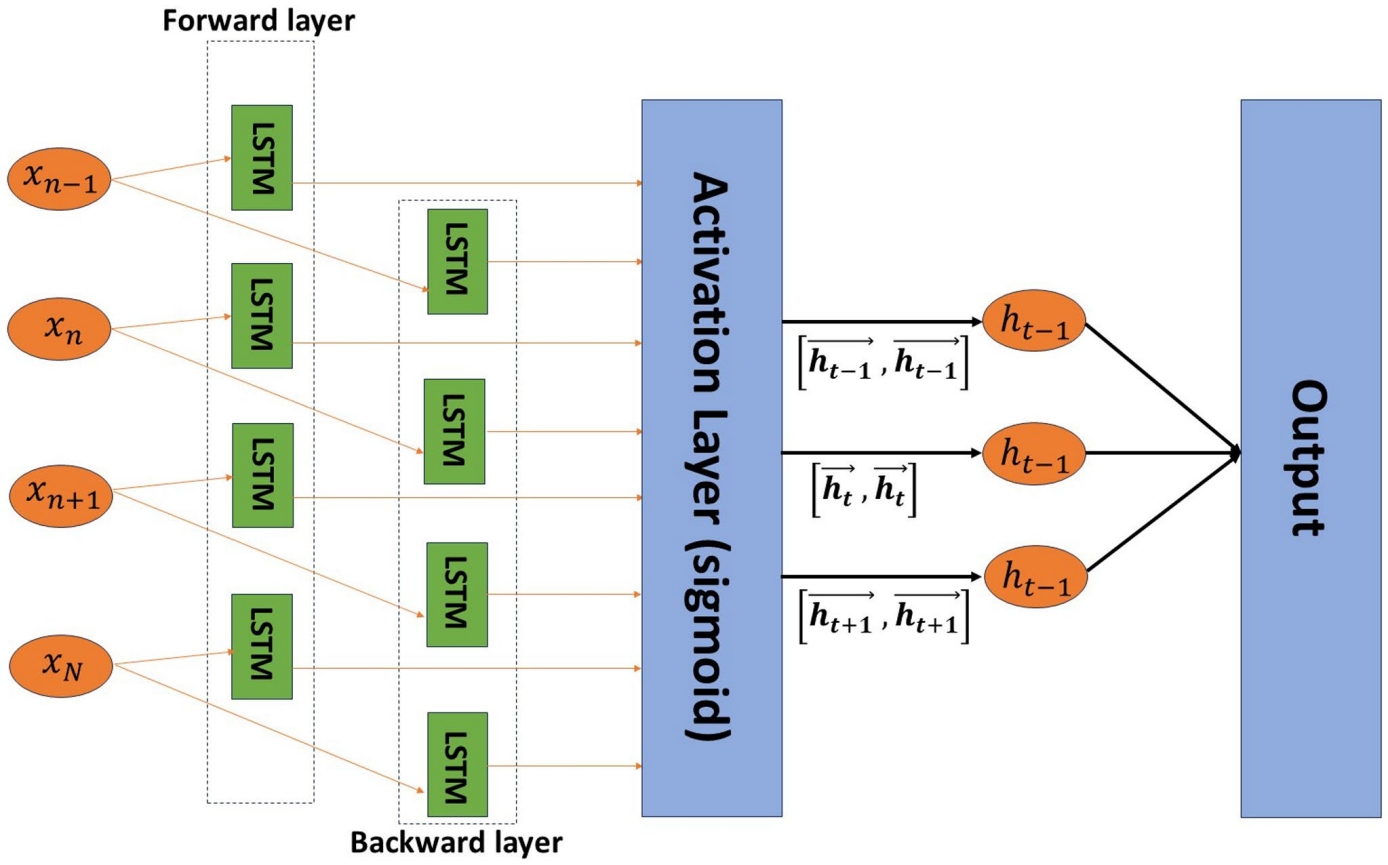
Dual-LSTM structure used in this study (Algarni et al., 2022).

To enhance the classification performance of LSTM, we propose the Dual-LSTM model. This classifier is trained with optimally selected features and is designed for emotion classification. In this paper, we use the developed Dual-LSTM for sentiment detection, which is a DL algorithm that processes the input sequence in both normal chronological order and reverse chronological order through two separate networks. As illustrated in Figure 5, the outputs of these networks are consecutive in each time step. The Dual-LSTM’s stacked layer architecture enables it to capture both forward and backward information about the sequence at each time step, resulting in high classification accuracy.

## 3. Results

### 3.1. The DEAP dataset

The proposed ER method was validated using the widely-used DEAP dataset for ER. This dataset consists of recordings from 32 participants while watching a music video. This dataset comprises recordings from 32 participants while they watched a music video. The dataset consists of 40 videos carefully selected to represent varying levels of arousal, valence, liking/disliking, dominance, and familiarity. The levels of arousal and valence were measured using numerical values ranging from 1 to 9. The dataset was recorded with a high EEG frequency of 512 Hz. Table 1 presents a summary of the key information about the dataset. In this study, the preprocessed data files were utilized, with each participant’s file containing two matrices: data and labels (as shown in Table 1). The dataset is divided into two primary matrices: the numerical data matrix and the label set. The data matrix has dimensions of 40 × 40 × 8,064 (video/trial × channel × data), encompassing channel and video data. The label set has dimensions of 4 × 40, representing the experiences of potency, arousal, mastery, and liking. The dataset was preprocessed using a band-pass filter with a frequency range of 4–45 Hz, and the EEG signals were down-sampled to 128 Hz (George et al., 2019).

**Table 1:** Brief description of DEAP database.

Description (description of parameters)	Value
Number of participants	32
The number of channels in EEG	32
Number of videos per participant	40
Sampling frequency before preprocessing	512
Sampling frequency after preprocessing	128
The number of tags in the database	4
The name of the tags	Arousal, valence, liking/disliking, dominance andfamiliarity
Number of data for each tag	Number of data for each tag
Data dimensions for each participant	
The number of tags in the database	40 × 40 × 8,064

### 3.2. Parameters

This paper applies the proposed Dual-LSTM DL neural method with appropriate settings to classify the preprocessed EEG data of 32 participants from the DEAP dataset who watched 40 videos, with the objective of identifying the emotions evoked by the videos. The Dual-LSTM model is particularly advantageous when considering the entire time sequence at each time step in the signal, as it effectively captures long-term dependencies between time steps. The Dual-LSTM model utilizes two layers of LSTM, operating in both forward and reverse modes for learning. For emotion detection in the proposed method, a five-layer classifier was trained, and the following parameters were employed:

- The maximum number of epochs was set to 35, allowing the model to perform 35 weight update iterations. The weight updating is performed using informative data.
- The mini-batch size was set to 80.
- An initial learning rate of 0.01 was used to enhance training speed by increasing the learning rate in the initial cycles. The gradient threshold was set to 1 to balance the training process by keeping gradients at a reasonable level.
- The “Plots” option was set to “Training Progress” to generate graphs illustrating the actual progress of the training process as the iterations increment.

Table 2 summarizes the parameters and settings of the classifier. The random search method involves selecting a random sample of data and testing the performance of the proposed model using randomly selected parameter sets. One of the main challenges of this approach is to identify an accurate classification method with parameters such as the learning rate and number of hidden layer units with significant impacts on the accuracy of the classifier. For instance, a low learning rate value can result in slow convergence, while a high value can lead to erratic and unstable performance. The number of hidden layer units also influences the fit of the model. Training dynamics are influenced by class size. A large batch size can result in poor generalization and increased memory requirements during training, while a small batch size can lead to convergence on training data. In this study, a reliable heuristic random search algorithm is employed to identify optimal parameter values that balance the performance and computational efficiency of the Dual-LSTM model. To train the model, we utilized the ADAM optimization algorithm which is a faster alternative to stochastic gradient descent methods commonly used in deep learning. ADAM combines the advantages of the AdaGrad and RMSProp algorithms, making it effective for handling sparse gradients and noisy data. This optimizer provides computational efficiency for training the Dual-LSTM model, making it suitable for large data or parameter sets. We used 70% of the total data for training the Dual-LSTM which consisted of 896 records of the feature matrix, while the remaining 30% (384 records) were used for testing. The number of features extracted from each EEG signal is shown in Table 3.

**Table 2:** Used classifier settings.

Parameter	The amount of
Educational rate	0.01
Optimization method	ADAM
The highest number of ipak	350
The smallest package size	80
The number of hidden units	100
Gradient threshold	1
Executive environment	Automatically
Hidden layers	1*5
The length of the sequence	The longest
shuffle	Once

**Table 3:** Features extracted from each EEG signal data channel.

Feature type	Features
Statistical features	Skewness, curve elongation, entropy, mean, variance, signal energy, normalized signal energy, Shannon energy
Other features	Hurst exponent

### 3.3. Evaluation criteria

The performance evaluation of this model involved assessing multiple criteria, including accuracy, recall, precision, and specificity. These criteria are quantified using Equation 3, as depicted below:


(3) $$\begin{array}{l} \mathrm{Accuracy}=\frac{TP+TN}{TP+FN+FP+TN}\\ \mathrm{Recall}=\frac{TP}{TP+FN}\\ \mathrm{Precision}=\frac{TP}{TP+FP}\\ \mathrm{Specificity}=\frac{TN}{TN+FP} \end{array}$$

In the Equation (3), TP, TN, FN, and FP are true positive, true negative, false negative, and false positive, respectively.

### 3.4. ER results with LSTM and Dual-LSTM

To assess the proposed method, we first evaluate each emotion individually. Next, we analyze the collection of all emotions as input for classification. We assess the accuracy and ROC criteria separately in all four states: arousal, capacity, liking/disliking, dominance, and familiarity, both before and after FS. Additionally, we compare the proposed classifier as recurrent DL classifier with the conventional LSTM classifier.

Table 4 shows the results of ER classification using statistical features, Hurst features, a combination of Hurst and statistical features, and FS method with LSTM and Dual-LSTM. As presented in Table 3, the use of LSTM in FS mode outperforms the non-FS mode. The results of using Dual-LSTM demonstrated a noticeable enhancement compared to the use of LSTM alone. This superiority can be attributed to the presence of memory and forgetting units in Dual-LSTM, which operate in a round-trip fashion. Finally, a combination of Hurst and statistical and Dual-LSTM led to the best performance for ER using the EEG signals. In Table 5, the results of proposed method was compared with previous ones.

**Table 4:** Comparison the results of different algotithms in this study.

Method	Accuracy	Sensitivity	Precision	Specificity
Statistical features - LSTM	89.13	79.06	79	92.64
Hurst features - LSTM	89.17	79.38	80.78	92.64
Hurst and statistical features - LSTM	93.71	88.02	88.58	95.63
FS - LSTM	94.05	88.67	89.45	95.92
Statistical feature – Dual-LSTM	90.05	80.01	82.25	92.96
Hurst features – Dual-LSTM	94.95	90.1	90.92	96.50
Hurst and statistical features - Dual-LSTM	96.83	94.04	94.27	97.88
Fs - Dual-LSTM	98.73	97.8	97.6	98.9

**Table 5:** Comparison the results of this study and previous studies.

Method	Accuracy
LSTM (Xing et al., 2019)	81.1 (valence), 74.38 (arousal)
LSTM-CNN (Yin et al., 2021)	90.45 (valence), 90.60 (arousal)
MLP (Thammasan et al., 2017)	64.1 (valence), 73 (arousal)
DNN (Pandey and Seeja, 2022)	62 (valence), 63 (arousal)
SVM (Girardi et al., 2017)	56 (valence), 60.4 (arousal)
LSTM-RNN (Alhagry et al., 2017)	85 (valence), 85.4 (arousal)
BI-LSTM (Algarni et al., 2022)	99.45 (valence), 96.87 (arousal), 99.68 (liking)
This study	98.73

The proposed method was evaluated by assessing individual emotions separately, followed by analyzing the collective set of emotions as input for classification. The accuracy and ROC criteria were assessed for four states: arousal, capacity, liking/disliking, dominance, and familiarity, both before and after applying the FS method. Additionally, the proposed classifier, a recurrent DL classifier, was compared to the conventional LSTM classifier.

**Figure 6:**
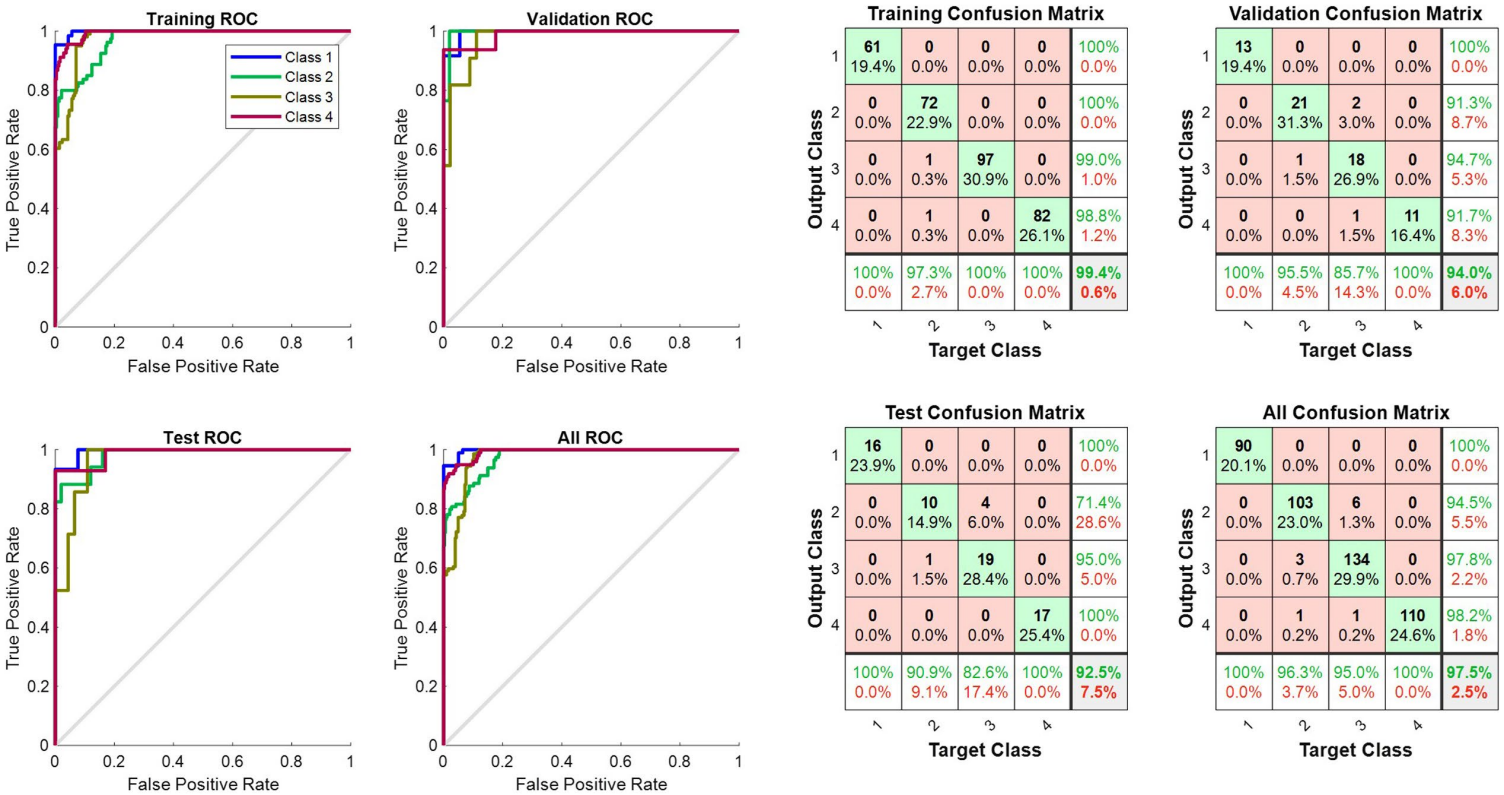
The ROC curve and confusion matrix for ER using the FS method on Hurst and statistical features and using the Dual-LSTM.

Table 4 presents the results of ER classification using different algorithms, including statistical features, Hurst features, a combination of Hurst and statistical features, and the FS method with LSTM and Dual-LSTM. As shown in Table 4, employing LSTM in FS mode outperformed the non-FS mode. Moreover, the results of using Dual-LSTM showed a significant improvement compared to using LSTM alone. This enhancement can be attributed to the presence of memory and forgetting units in Dual-LSTM, which operate in a bidirectional manner. Finally, combining Hurst and statistical features with Dual-LSTM yielded the best performance for ER using EEG signals. Figure 6 shows the ROC curve and confusion matrix for ER using FS method on Hurst and statistical features and using Dual-LSTM. As depicted in Figure 6, the proposed method could classify most emotion types with high accuracy rate.

Table 5 provides a comparison between the results of the proposed method in this study and previous studies. The accuracy achieved in this study (98.73%) outperformed most of the previous studies.

## 4. Discussion

The recognition of emotions using EEG signals presents several challenges due to their nonstationary nature and complexity. This paper proposed an effective solution for ER based on DL. The proposed deep learning approach significantly enhances the accuracy of ER using EEG signals. In this study, DL was utilized for ER with EEG signals, employing a DL-based classification approach using the LSTM classifier. The LSTM classifier relied on key features, such as Hurst and statistical features, which were extracted from preprocessed input signals. To select the most distinctive features for emotion recognition and classification, a filter-based FS method was employed. The proposed method consisted of several stages, including preprocessing, feature extraction, dimensionality reduction through feature selection, and classification. The combined use of multiple feature series led to improved recognition accuracy, with the Dual-LSTM model outperforming the LSTM model in FS mode. Table 4 displays the results of ER using FS and other features, including statistical and Hurst features, based on LSTM and Dual-LSTM classification. The results demonstrated that the feature selection algorithm enhanced the classification performance, with Dual-LSTM achieving higher accuracy, sensitivity, precision, and specificity compared to LSTM. Dual-LSTM captures information from both past and future time steps, resulting in a better understanding of the sequence dynamics. In contrast, LSTM processes the input sequence only in the forward direction. Table 5 compared the accuracy of the proposed model with other DL and ML methods using the DEAP dataset. The results showed a significant improvement over previous models, thanks to the adoption of an improved approach compared to traditional LSTM models. However, differences in feature extraction techniques, classification methods, and parameters led to varying classification accuracies, despite utilizing the same dataset. The use of an appropriate FS method is crucial for enhancing the model’s performance. Compared to prior research using RNN and LSTM, the proposed method yielded exceptional outcomes. It is worth noting that earlier studies mainly focused on valence and arousal, while this study successfully classifies all four emotions.

## 5. Conclusion

This study employs Dual-LSTM deep neural networks for ER. In the proposed method, after extracting the statistical features and Hurst features, dimension reduction was performed using the proposed method in this study. The Dual-LSTM neural classifier was then used for classification, and its performance was compared with other classifiers including LSTM using the accuracy, precision, recall, and specificity criteria. The results demonstrate that the use of Dual-LSTM coupled with dimension reduction of the statistical and Hurst features outperforms other methods. Additionally, the feature extraction and FS stages based on LSTM demonstrated better results compared to the other methods, likely due to their data-driven nature.

## Data availability statement

The original contributions presented in the study are included in the article/supplementary material, further inquiries can be directed to the corresponding author.

## Ethics statement

Ethical approval was not required for the study involving humans in accordance with the local legislation and institutional requirements. Written informed consent to participate in this study was not required from the participants or the participants’ legal guardians/next of kin in accordance with the national legislation and the institutional requirements.

## Author contributions

All authors listed have made a substantial, direct, and intellectual contribution to the work and approved it for publication.
